# Heterogeneity of *TP53* Mutations and P53 Protein Residual Function in Cancer: Does It Matter?

**DOI:** 10.3389/fonc.2020.593383

**Published:** 2020-10-28

**Authors:** Paola Monti, Paola Menichini, Andrea Speciale, Giovanna Cutrona, Franco Fais, Elisa Taiana, Antonino Neri, Riccardo Bomben, Massimo Gentile, Valter Gattei, Manlio Ferrarini, Fortunato Morabito, Gilberto Fronza

**Affiliations:** ^1^Mutagenesis and Cancer Prevention Unit, Istituto di Ricerca e Cura a Carattere Scientifico (IRCCS) Ospedale Policlinico San Martino, Genoa, Italy; ^2^Molecular Pathology Unit, IRCCS Ospedale Policlinico San Martino, Genoa, Italy; ^3^Department of Experimental Medicine, University of Genoa, Genoa, Italy; ^4^Department of Oncology and Hemato-oncology, University of Milan, Milan, Italy; ^5^Hematology, Fondazione Cà Granda IRCCS Policlinico, Milan, Italy; ^6^Clinical and Experimental Onco-Haematology Unit, Centro di Riferimento Oncologico, I.R.C.C.S., Aviano, Italy; ^7^Hematology Unit, Azienda Ospedaliera (AO) di Cosenza, Cosenza, Italy; ^8^Unità di Ricerca Biotecnologica, Azienda Sanitaria Provinciale di Cosenza, Aprigliano, Italy; ^9^Department of Hematology and Bone Marrow Transplant Unit, Augusta Victoria Hospital, Jerusalem, Israel

**Keywords:** *TP53* mutations, chronic lymphocytic leukemia, clinical impact, P53 protein function, reactivation of P53

## Abstract

The human *TP53* locus, located on the short arm of chromosome 17, encodes a tumour suppressor protein which functions as a tetrameric transcription factor capable of regulating the expression of a plethora of target genes involved in cell cycle arrest, apoptosis, DNA repair, autophagy, and metabolism regulation. *TP53* is the most commonly mutated gene in human cancer cells and *TP53* germ-line mutations are responsible for the cancer-prone Li-Fraumeni syndrome. When mutated, the *TP53* gene generally presents missense mutations, which can be distributed throughout the coding sequence, although they are found most frequently in the central DNA binding domain of the protein. *TP53* mutations represent an important prognostic and predictive marker in cancer. The presence of a *TP53* mutation does not necessarily imply a complete P53 inactivation; in fact, mutant P53 proteins are classified based on the effects on P53 protein function. Different models have been used to explore these never-ending facets of *TP53* mutations, generating abundant experimental data on their functional impact. Here, we briefly review the studies analysing the consequences of *TP53* mutations on P53 protein function and their possible implications for clinical outcome. The focus shall be on Chronic Lymphocytic Leukemia (CLL), which also has generated considerable discussion on the role of *TP53* mutations for therapy decisions.

## Introduction

The human tumour suppressor gene *TP53*, located at 17p13.1 locus, encodes a 393 amino acid-long protein, which was discovered in the 80s of last Century within a complex containing the viral SV40 large T antigen (1–3). Initially misclassified as an oncogene, because of the isolation of mutant cDNA clones capable of inducing cell transformation, the wild type (WT) *TP53* gene was eventually classified as tumour suppressor, upon the definite demonstration of its capacity of inhibiting the growth and the oncogenic transformation of cells in culture (4). Concurrently, somatic *TP53* mutations were identified in tumours (5, 6) and germ-line *TP53* mutations were described in the Li-Fraumeni syndrome (LFS), the well-known hereditary cancer predisposition disorder (7).

**Figure 1:**
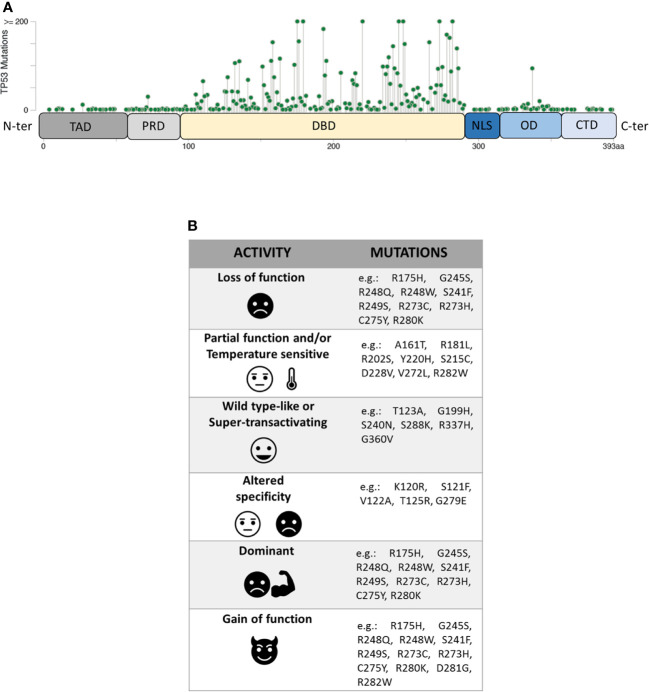
**(A)** Domain organization of P53 protein (TAD, transactivation domain; PRD, proline-rich domain; DBD, DNA-binding domain; NLS, nuclear localization signal; OD, oligomerization domain; CTD, C-terminal domain) and distribution of *TP53* missense mutations from the cBioPortal online tool (TCGA) (8, 9). Missense mutations location (green signs) throughout the P53 protein is shown according to the frequency and the position of the amino acid hit. **(B)** Heterogeneity of mutants P53 with examples of the corresponding *TP53* mutations.

The P53 protein consists of different functional domains including mainly a N-terminal transactivation domain (residues 1–61, TAD), a central DNA binding domain (residues 94–290, DBD), an oligomerization domain (residues 325–356, OD) and a C-terminal domain that regulates the DNA binding (residues 357–393, CTD) (**Figure 1A**) (10). While the TAD domain interacts with components of the transcription machinery, the OD and the DBD domains are necessary for the formation of the P53 tetramer which interacts with specific DNA target sequences, called P53 response elements (P53 REs) that are comprised of two degenerate decameric sequences [Pu (Purine)-Pu-Pu-C-A/T-AT-G-Py (Pyrimidine)-Py-Py] separated by a variable spacer (11). To complicate the scenario further, different isoforms of P53, resulting from the usage of alternative promoters and splicing sites, or alternative initiation sites of translation, have been recently described (12, 13).

P53 is a transcription factor (TF) which can be induced by endogenous and exogenous stresses (e.g. oncogenes and UV radiation); these stresses activate signals, which operate *via* post-translational modifications on P53 protein (e.g. phosphorylations, acetylations), and cause release of P53 from the mortal embrace with MDM2 protein, eventually leading to P53 activation (14). P53 protein can then enter the nucleus where it induces the expression of a plethora of target genes (15). However, increasing observations are reporting certain “non-transcriptional functions” of P53, that can contribute to tumour suppression activity (16).

## *TP53* Mutations Heterogeneity

Unlike other tumour suppressors, the *TP53* gene is mostly altered by missense mutations, mainly involving the portion coding for the DBD domain of the P53 protein. Within this region, eight amino-acid substitutions (i.e. R175H, G245S, R248Q, R248W, R249S, R273H, R273S, and R282W), called hotspot mutations, characterize ~27% of all mutant P53 proteins identified in human cancers. However, the spectrum of missense mutations is extremely broad (**Figure 1A**) (8, 9, 17, 18) and varies in the different tumour types.

Mutant P53 proteins have been primarily categorized as “contact” or “structural”, depending on whether substituted amino-acid was directly involved in the interaction with DNA (e.g. R273H) or caused a global effect on the protein structure (*e.g.* R175H) (19, 20). Over the last 25 years, a large amount of experimental data on the functional impact of different amino-acid substitutions has been generated and different models have been used to explore the never-ending facets of the corresponding mutant proteins. One of these models is the yeast *S. cerevisiae*, exploiting the fact that P53 can act as TF also in this organism by binding P53 REs located upstream a reporter gene (21, 22). The assay, originated as a Functional Analysis of Separate Alleles in Yeast to study the *TP53* status in somatic tumour and blood cells, has been unceasingly modified and upgraded in order to explore additional functions of P53 mutants (23–26). In 2003 Ishioka’s group tested the functionality of more than 2,000 different P53 single amino-acid substitutions expressed in yeast (27). Their work, along with that of others (23–26), led to a re-classification of the *TP53* mutations based on their effects on P53 function generating the following categories: i) loss of function (LOF), ii) partial function and/or temperature sensitive (PF, TS), iii) WT-like or super-transactivating (WT-L, ST), iv) with altered specificity (AS) (i.e. active or partially active on some targets but inactive on others), and v) dominant-negative (DN), based on the ability to inhibit WT protein in a heterozygous condition. However, mutant P53 proteins might be classified also as gain of function (GOF) related to the acquisition of novel oncogenic activities, not shared with the WT protein (**Figure 1B**). This latter property is mainly based on the ability of the mutant P53 to interact with other TFs or with chromatin-modifying complexes, altering the cellular transcriptional profile (28–32). Therefore, mutant P53 proteins appear to constitute a functional rainbow (**Figure 1B**) (33).

Recently, two seminal papers confirmed the relevance of the heterogeneity of mutants P53 in terms of Relative Fitness Score (RFS) in *in vitro* cultures (34, 35). RFS has been used as an indicator of the functional impact of *TP53* mutations in terms of selective growth; specifically, a high RFS indicates a higher fitness of the *TP53* variant with preferential expansion within mixed cultured cells in competition with other *TP53* variants, whereas a low RFS pinpoints preferential depletion in the same experimental condition. Moreover, Kotler et al. (34) showed that the loss of the anti-proliferative function of WT P53 largely correlates with the occurrence of cancer-associated *TP53* mutations, and that selective GOF properties may further favour specific mutants P53 *in vivo*. An enhanced cellular fitness was also confirmed in association with the loss of WT P53 function or the DN effect associated to specific *TP53* mutations (35).

## Impact of *TP53* Mutations Heterogeneity: From Mice to LFS Clinical Condition

A clear evidence of the different impact of *TP53* mutations at the organism level came from the studies with *Trp53* knock-in mice. In mice, the introduction of the R172H mutation (corresponding to the human R175H hotspot) at the germ-line level generates a tumor phenotype similar to that observed in *Trp53* null mice, but with a much higher rate of metastasis (36). The GOF activity of R172H mutation was demonstrated to be associated with the functional inactivation of P63 and P73 TFs (37). In contrast, the R172P mutation in mice (corresponding to the human R175P, PF mutation) caused a delayed tumorigenesis rate with absence of chromosomal instability (38). These findings have been paralleled by observations made in patients affected by LFS, showing that the age of first tumor onset and the spectrum of observed tumors are dependent on the type of *TP53* mutation (39).

The associations of the genotype with clinical outcome was explored in carriers of *TP53* germ-line mutations also using a functional classification of the mutant P53 based on the quantification of their transactivation potential and DN effect in a yeast reporter assay. The analyses revealed that P53 proteins severely deficient in transactivation capability were more frequently associated with more severe cancer proneness syndromes (e.g. LFS) (40), whereas a further classification of these alleles, based on DN effects, did not distinguish clinical subclasses (41).

## Impact of *TP53* Mutations Heterogeneity in Somatic Cancers

Although the majority of the studies on the prognostic and predictive role of *TP53* status in human cancers distinguish between patients harboring WT *versus* mutant proteins, some evidence favors a categorization of *TP53* mutations since different mutant P53 proteins can have different biologic effects. Poeta et al. (42) proposed the distinction between “disruptive” and “non-disruptive” *TP53* mutations; while disruptive mutations likely lead to a complete loss of activity of the P53 protein, non-disruptive mutants can encode proteins which retain some of the original functions. This classification was used to stratify patients with *TP53* mutations in head and neck squamous cell carcinoma (42), advanced Non-Small Cell Lung Cancer (43), breast and ovarian tumors (44, 45) and esophageal squamous cell carcinoma (46). However, the association between the type of *TP53* mutations and prognosis was significantly variable in the different cancers.

Recently, Dutta et al. (47) analyzed data from 1,537 patients with Acute Myeloid Leukemia (AML) in order to determine a correlation between *TP53* mutations and clinical outcome. *TP53* mutations have been classified according to (i) their impact on protein structure (disruptive *versus* non-disruptive), (ii) an evolutionary action score that takes into account the evolutionary sensitivity to sequence variation and amino-acid conservation (48) and (iii) the RFS (34). Only the RFS was capable of distinguishing among AML patients with a significantly different overall survival and event-free survival. All these observations complicate the scenario and the definition of the events which are drivers of the disease pathogenesis.

## Impact of *TP53* Mutations Heterogeneity: Restoration of WT P53 Functionality

Mutant P53 protein has been considered a promising target for the development of new anticancer strategies and, in the last two decades, several molecules have been developed with the aim of reinstating the WT function or eliminating the mutant P53 accumulated in cancer cells (49). Upon re-acquisition of its original WT properties, the P53 protein should become transactivation competent (50), and trigger an efficient apoptotic response following treatments to which the tumor cells are normally refractory. PRIMA-1 and its methylated derivative PRIMA-1^Met^ (also named APR246) are the most widely studied molecules on which phase I/II clinical trials are in progress (51). These molecules were found capable of reconstituting the specific DNA binding capacities to different mutant proteins (e.g. R273H, R175H) and of inducing significant apoptosis in cancer cells carrying a mutant P53 protein (52). Another molecule, RITA, which interacts with P53 and inhibits its binding to MDM2, induces a P53-dependent gene transcription and cell death (53). Beside these, many others molecules, which target the interaction of WT P53 with negative regulators (e.g. Nutlins) or with the mutant P53 (e.g. CP31398), have been investigated, some of them being currently tested in clinical trials (53, 54).

A different approach is based on the potential inhibition of the GOF activities, obtained by promoting mutant P53 protein degradation. Since mutant P53 is stabilized by the heat shock protein HSP90, usually over-expressed in cancer cells (55), several HSP90 inhibitors, such as 17-AAG or Ganetespib, have been tested as anticancer molecules and their ability to trigger mutant P53 degradation has been demonstrated (56). Also Histone Deacetylase inhibitors (HDAC), such as SAHA, can induce the degradation of the mutant P53, restraining tumor growth *in vivo* (56, 57). Lastly, a role of autophagy to trigger mutant, but not WT, P53 deprivation has been shown in different cancer cells (58–60), identifying the modulation of autophagy as an emerging strategy for cancer therapy (61, 62).

## Not Just a Question of *TP53* Mutations

P53 total inactivation in human cancer cells is frequently caused by the alterations of both alleles, comprising the allelic loss due to deletion of the short arm of the chromosome 17 [del(17p)], and the concomitant mutation of the other allele. It is of note that Donehower et al. (63) performing a comprehensive assessment of the P53 pathway involvement in 32 cancers from The Cancer Genome Atlas, demonstrated the loss of the second allele in 91% of the cases with *TP53* mutations. In addition, in heterozygous murine tumours carrying the hotspot GOF allele R248Q, the loss of the remaining WT *TP53* allele was a necessary prerequisite for the stabilization of the mutant P53 and for the GOF properties to become evident *in vivo* (64). These observations suggest that a given *TP53* mutation must operate in a specific cellular context to show its biological consequences (65).

## Clinical Impact of *TP53* Alterations: The Example of Chronic Lymphocytic Leukemia

Chronic Lymphocytic Leukemia (CLL) is the most common leukemia in the Western countries, characterized by the clonal expansion of CD5+ B cells in peripheral blood, lymph-nodes and bone marrow. CLL clinical course is highly heterogeneous (66), ranging from decades of survival with no need for treatment, to a rapid disease progression with the requirement for an early treatment (67). Such a scenario likely reflects the cellular and molecular heterogeneity of the disease. CLL cases present specific karyotype aberrations, the most frequent being 13q- (~55%), 11q- (~15%) 17p- (~8%) and +12 (~15%), which correlate with a different disease course and outcome (68). In addition, gene mutations (e.g. *TP53*, *SF3B1*, *BIRC3*, and *NOTCH1*) have been reported (69), which, again, may influence the disease course and outcome. B cell receptors (BCR) features expressed by the leukemic cells also dictate the subsequent patient fate as demonstrated by the fact that patients with somatically mutated IGHV genes in the leukemic cells have a better clinical course and outcome than the patients in whom such genes are not somatically mutated; it is generally assumed that stimulation of the leukemic cells by self or exogenous antigens may promote clonal expansion (70). This notion is supported by the observation that inhibitors of the BCR-dependent signal transducing pathway are efficient treatments for CLL (70). In addition, different CLL patients that share the same BCR have similar clinical courses (71). Finally, patients with complex karyotypes, detected by chromosome G-banding, may have a dire prognosis, even in the era of new drugs (72).

P53 dysfunction has certainly a role in the clinical evolution of CLL (73). The incidence of *TP53* mutations is low at diagnosis (<10% of patients), although it rises in cases with progressive disease and reaches approximately 40% in refractory CLL (73–77). Furthermore, there is evidence that CLL patients with *TP53* dysfunction [measured as del(17p) and/or *TP53* mutations] progress more rapidly to stages requiring treatment. Together, these considerations indicate that *TP53* alterations facilitate clonal expansion and disease progression irrespective of the impact they may have on therapy (78). The presence of a P53 dysfunction has a definite negative impact on the effect of chemo-immunotherapy, whereas such impact appears to be less pronounced in patients treated with BCR inhibitors (e.g. Ibrutinib or Idelalisib) or with apoptosis inducers (e.g. Venetoclax). Because of this, *TP53* mutational screening for all patients before therapy start is recommended by the European Research Initiative on CLL group (ERIC) to avoid treatment protocols that are ineffective in patients with P53 dysfunction (79).

Detection of a del(17p) or of a *TP53* mutation is generally assumed to be a sufficient indication for a P53 dysfunction. CLL patients with del(17p) carry a *TP53* mutation in 80% to 90% of the cases, and ~60% of patients with *TP53* mutations also harbor del(17p), as detected by FISH. Even in the absence of del(17p), the presence of a *TP53* mutation appears to be more frequent in patients with a poor prognosis and a higher genetic complexity (80, 81). Moreover, CLL sub-clones carrying specific *TP53* mutations can be positively selected upon treatment, ultimately becoming the prevalent expansion of an initially minor mutant component (69, 82–84).

## Dealing With Complexity in CLL

The identification of molecular biomarkers together with certain clinical features of the disease may dictate the choice of treatment in CLL (85). Since a P53 dysfunction is the strongest predictor of chemo-refractoriness, the assessment of *TP53* status is the first, and possibly most important, decisional node in the first-line treatment algorithm. Indeed, the presence of P53 dysfunctions prevents the use of chemo-immunotherapy in favour of BCR inhibitors or Venetoclax (78). However, although such drugs have improved the poor efficacy of chemo-immunotherapy in patients with del(17p) and/or *TP53* mutations (86), all these treatments still pose some challenges in these patients. Furthermore, the real influence of a gene dosage effect [e.g. presence of del(17p) *versus* presence of both deletion and a TP53 mutation] in patients treated with the new drugs has still to be clarified (78).

**Figure 2:**
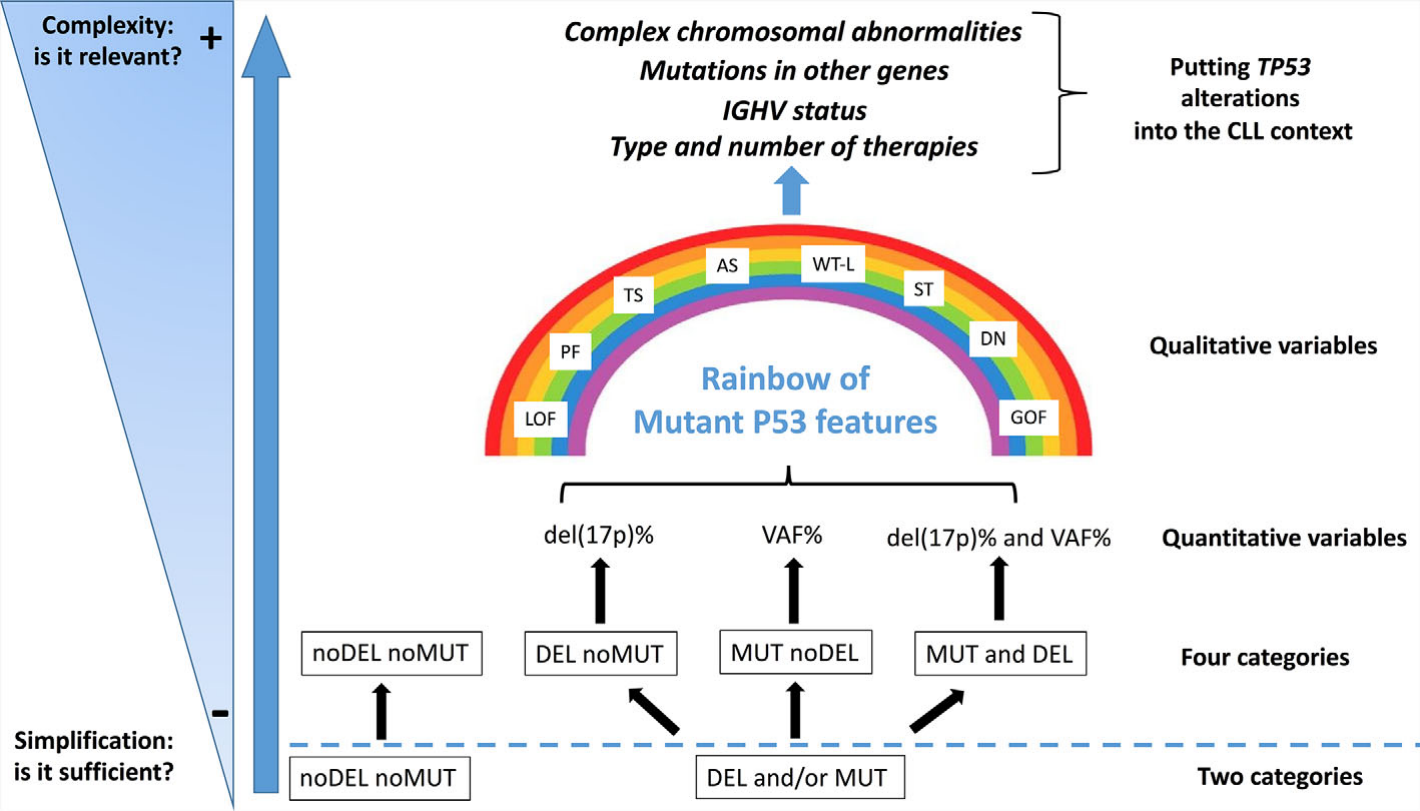
Del(17p) (DEL), detected by FISH, and/or *TP53* mutation (s) (MUT), detected by DNA sequencing, as indicators of a P53 dysfunction. The present classification may represent an oversimplification of the conditions leading to an impaired P53 function, both in terms of quantity [del(17p) % or VAF, Variant Allele Frequency %] and quality (i.e. P53 mutant protein functionality: LOF, loss of function; PF, partial function; AS, altered specificity; TS, temperature sensitive; WT-L, wild-type like; ST, super-transactivating; GOF, gain of function; DN, dominant negative). Moreover, other molecular features (e.g. mutated IGHV *versus* un-mutated IGHV; SF3B1, BIRC3 and NOTCH1 mutations; 13q and 11q partial deletions) and the eventual previous patient management might influence the highly heterogeneous clinical course of the disease.

Although genomic technologies are changing the practice of onco-haematology, with improved detection of driver lesions, genomic data, generated through different technologies, each with its own sensitivity, are often considered not only interchangeable [i.e. equivalence between the presence of del(17p) and of a *TP53* mutation (*TP53*mut)], but are also subjected to oversimplification [i.e. equivalence between the presence of one *TP53* alteration (mutation or deletion) and of both alterations)]. Even though a binary simplification (P53 dysfunction *versus* no P53 dysfunction) can be considered clinically usable, the actual situation is potentially more complicated than estimated (87) and a more realistic situation diverging from a simple binary scenario (noDel/noMut *versus* Del and/or Mut) could be conceived and proposed for the clinical use in the future (**Figure 2**). Furthermore, the abundance of the single *TP53* alteration within the leukemic clone [i.e. Variant Allele Frequency (VAF) for a *TP53* mutation and percentage of del(17p) positive cells] may represent a factor of relevance. For example, while all identified *TP53* mutations were clonal with the Sanger sequencing method (VAF>10%), both clonal (VAF>10%) and sub-clonal (VAF<10%, as small as 0.3%) *TP53* mutations can be detected with the introduction of Next Generation Sequencing technologies. Nevertheless, this information has not entered into clinical practice yet, although it may contribute to provide information on the effective P53 function in the leukemic clone and also on its potential prospective evolution. The last update of the guidelines released by ERIC still consider that clinical decisions should be taken based on the presence of a clonal *TP53* mutation.

The percentage of del(17p) positive cell may also represents an important variable as it appears that, among patients with del(17p), those with a higher percentage of cells carrying the deletion have shorter survivals (88). Another layer of complexity is related to the fact that not all mutant P53 proteins appear to have the same functional consequences, as previously described. Although these issues, which are related to the effective P53 function in a leukemic clone, have emerged as real biological and clinical problems, they have not been so far deeply investigated in CLL (65). Finally, it should be stressed that *TP53* alterations, although important, are not the sole alterations and should be considered together with other cytogenetic abnormalities which may occur concomitantly in the single patients and may affect *per se* the clinical course of CLL (**Figure 2**).

## Concluding Remarks

In conclusion, is the binary scenario compatible with the underlying complexity in CLL? While a simplified vision is important for deciding clinical strategies, new studies appear necessary for assessing whether further levels of complexity in CLL classification, can lead to a more precise patient stratification. In this context, it is likely that future studies will define whether patients with del(17p) and a *TP53* mutation might have a different clinical course from those who have only a *TP53* mutation or only del(17p). Furthermore, a patient with a partial function *TP53* mutation might show a different clinical course from those harbouring a complete loss of function *TP53* mutation, as suggested by our present observations and studies in other experimental systems (33, 89, 90). All of these aspects regarding P53 dysfunction may affect therapy and consequently deserve an evaluation, possibly more extended than that currently used.

## Author Contributions

All authors contributed to the article and approved the submitted version.

## Funding

This work was supported by: Associazione Italiana Ricerca sul Cancro (AIRC) Grant 5 x mille n.9980, (to MF, FM, and AN); AIRC I.G. n.14326 (to MF), n.15426 (to FF), and n. 5506 (to GF); AIRC and Fondazione CaRiCal co-financed Multi-Unit Regional Grant 2014 n.16695 (to FM); Italian Ministry of Health 5x1000 funds 2013 (to GF) 2014 (to GC, SZ, and AI), 2015 (to FF and GF) and 2016 (to FF, GC, and GF); Current Research 2016 to GF; and 2016 (to FF and GC); Compagnia S. Paolo Turin Italy project 2017.0526 (to GF) Italian Ministry of Health: Alleanza Contro il Cancro (Hematology network).

## Conflict of Interest

The authors declare that the research was conducted in the absence of any commercial or financial relationships that could be construed as a potential conflict of interest.
